# Hypoxic Training in Obese Mice Improves Metabolic Disorder

**DOI:** 10.3389/fendo.2019.00527

**Published:** 2019-08-08

**Authors:** Ru Wang, Shanshan Guo, Haili Tian, Yiru Huang, Qin Yang, Kewei Zhao, Chia-Hua Kuo, Shangyu Hong, Peijie Chen, Tiemin Liu

**Affiliations:** ^1^School of Kinesiology, Shanghai University of Sport, Shanghai, China; ^2^State Key Laboratory of Genetic Engineering, Department of Endocrinology and Metabolism, School of Life Sciences, Zhongshan Hospital, Fudan University, Shanghai, China; ^3^State Key Laboratory of Pharmaceutical Biotechnology, Nanjing University, Nanjing, China; ^4^Laboratory of Exercise Biochemistry, University of Taipei, Taipei, Taiwan

**Keywords:** hypoxic training, obesity, metabolomics, liver, metabolism

## Abstract

Hypoxic training has been reported to lower obesity morbidity without clear underlying mechanisms. This study investigates the effect of hypoxic training on metabolic changes, particularly, on liver metabolism of high fat diet (HFD)-induced obese mice. We compared the hypoxic training group with normoxic sedentary, normoxic training, and hypoxic sedentary groups. Body weight, fat mass, glucose tolerance and liver physiology were determined after 4 weeks intervention. In both normoxic training and hypoxic training groups, body weight was lower than the normoxic sedentary group, with less fat mass. Insulin sensitivity was improved after hypoxic training. Moreover, liver metabolomics revealed insights into the protective effect of hypoxic training on HFD-induced fatty liver. Taken together, these findings provide a molecular metabolic mechanism for hypoxic training.

## Introduction

The recent observation that in obese subjects adipose tissue becomes hypoxic and triggers inflammation and obesity-associated diseases (1) has generated inquiries as to the potential of oxygen therapy as a tool for weight management (2, 3). At the same time, there is research that indicates that hypobaric hypoxia and normobaric hypoxia can lead to weight loss and lower the risk of metabolic syndrome, respectively (4–6). While the mechanism underlying these observations is still unknown, combining hypoxia and exercise training might provide a cost-effective strategy for reducing body weight and improving metabolic health in obese humans.

Previously, hypoxic training was used to increase exercise performance of athletes. “Live high-train low” (normobaric hypoxia living and normoxic training) and “live low-train high” (normoxic living and hypobaric hypoxia training) were the most popular methods (7). It has been established that, long-term severe hypoxia exposure causes impairment of skeletal muscle and vascular endothelial function, and vascular hemodynamics (8, 9). At the tissue level, it was reported that acclimatization to hypoxia (10 days at 5,500 m) reduced the aerobic capacity of the rat skeletal muscle (gastrocnemius) through a fall in citrate synthase activity (10). Another study has shown that normobaric hypoxic (FIO_2_ = 15%) training enhanced the activation of satellite cells and angiogenesis of the thoroughbred horse skeletal muscle (9).

In obese subjects, the liver—an organ, critical to glycogenesis, glycogen storage, lipogenesis, fatty acid oxidation, lipolysis and decomposition of erythrocytes- is in a “hypermetabolic state.” Especially in obese subjects, endogenous metabolites are altered in response to nutrition consumption and energy expenditure. Therefore, metabolomics provides a potential platform to monitor changes in liver metabolites under hypoxic training. A metabolomics approach, to characterize variations in metabolite profile and identify biomarkers level with/without hypoxic training on obese subjects, would contribute to the dearth of literature.

To date, the combination of hypoxia and exercise has mainly been investigated in normal weight (BMI <25 kg/m^2^) or lean individuals. Very few studies have included obese subjects—those that have not focused on metabolic or body composition changes associated with hypoxic exercise. Specifically, in recent studies that included obese subjects, compared to non-obese subjects, the obese group reported greater reductions in serum reactive oxygen species (ROS) after oxidative training (11, 12).

The purpose of this study is to investigate the metabolic effects of hypoxic training on high fat diet (HFD)-induced obese mice. We hypothesize that, compared to normobaric hypoxia and normoxic training, hypoxic training would result in greater weight loss and changes in liver glucose and lipid metabolism in obese mice.

## Materials and Methods

### Animal Subjects

Four-weeks-old healthy male C57BL/6J mice were obtained from the Experimental Animal Center of Shanghai Second Military Medical University. All mice were housed under standard laboratory conditions (12 h on/off; lights on at 8:30 a.m.) and a temperature-controlled environment (22–24°C) with food and water available *ad libitum* in the SPF animal research center of Shanghai University of Sports (SYXK 2014-0002). All experiments were performed in accordance with the guidelines, established by Science Research Ethics Committee at the Shanghai University of Sports (No. 2015013) and approved by the Animal Care and Use Committee at the Shanghai University of Sports. Mice were fed with a HFD (Research Diet, #D12492; 60% kcal from fat, 5.24 kcal/g) beginning at 5 weeks old.

### Animal Model Preparation

After 13 weeks on HFD, the 18-weeks-old mice were randomly divided into four treatment groups: normoxic sedentary (S), normoxic training (NT), hypoxic sedentary (H), and hypoxic training (HT). While the mice were divided into four treatment groups, they were weight-matched. Treatments lasted for 4 weeks.

### Training and Hypoxic Intervention

#### Treadmill Training Protocol

Treadmill training was performed as previously described with modifications (13). Briefly, at 18 weeks of age, mice were acclimated to treadmill training for 3 days. From week 19 onwards, mice trained 6 days/week (Monday–Saturday) with a daily run time of 90 min. Each run began with 8 min at a speed of 6 m/min. From minute 9 to minute 30, the speed was gradually increased (1 m/min increase every 3 min) until a maximum speed of 14 m/min was reached at minute 30. Mice were kept running at 14 m/min from minute 30 to minute 90.

#### Establishment of Hypoxic Environment

The TSE PhenoMaster hypoxia metabolism warehouse was used to establish a moderate and constant hypoxic experimental environment. Oxygen concentration was set at 14.7% (although actual oxygen concentration ranged from 14.4 to 14.7% during the experiment) based on the well-established observation that altitude stress is induced at 3,000 m above sea level (~14.4% oxygen concentration) and our pre-experimental data showed that humans behaved normally in food consumption and other daily activities without any adverse reaction when oxygen concentration was below 14.7%. Intervention bouts were performed 8 h/day, 6 days/week (Monday–Saturday).

### Glucose Tolerance Tests

After an overnight fasting, 22-weeks-old mice were treated with intraperitoneal (i.p.) injections of 2 g/kg D-glucose. Blood glucose was measured from tail blood using a glucometer (Roche) at serial time points as indicated in figures. The areas under the curve (AUCs) were calculated using trapezoidal integration.

### Insulin Tolerance Tests

After a 4-h fasting to empty the stomach, 22-weeks-old male mice received i.p. injections of insulin (1.0 U/kg). Blood glucose level was measured from tail blood as described above. The areas under the curve (AUC) were calculated using trapezoidal integration.

### Immunohistochemistry

#### Oil Red O Staining

To compare size and density of lipid droplets in mice liver, liver was stored in 4% paraformaldehyde (Wuhan Google Biotechnology) for more than 12 h, then embedded in OCT (Sakura), cut on a cryotome (E, Thermo) at 8–10 μm thickness and preserved in −20°C freezer. Snap-frozen liver sections were fixed with 4% paraformaldehyde, washed three times in phosphate buffer saline (PBS), then incubated with oil red O (Wuhan Google Biotechnology) for 10–15 min, following three times washing in PBS. Sections were stained with Harris (Wuhan Google Biotechnology) right after oil red O-staining and finally washed with flowing water.

#### Hematoxylin and Eosin Staining

To assess general morphology of liver, liver was stored in 4% paraformaldehyde (Wuhan Google Biotechnology) for more than 12 h, then the tissues were processed routinely for paraffin embedding, and 4-μm-thick sections were cut and placed on glass slides. The paraffin-embedded sections were dewaxed with xylene, washed by gradient ethanol to water, then incubated with hematoxylin and eosin (Servicebio) for 5 min, and sealed after conventional ethanol dehydration. Finally, sections were analyzed under a Nikon light microscope at the indicated magnification.

### Metabolite Analysis by NMR

#### Preparation of Liver Samples for NMR Analysis

Approximately 55 mg of each sample was lysed in 600 μl ice-colded 80% methanol by Tissue-lyzer (QIAGEN Tissuelyzer, Germany) with stainless-steel beads (20 Hz for 90 s) (14). The lysate was transferred to new tubes and ultraphonic for 10 times (for a duration of 60 s each time with a 60-s interval between times) on ice, and the supernatant was collected by centrifugation (4°C, 11,180 g, 10 min). The pellet was extracted two more times following the same procedure. Pooled supernatants were centrifuged for 10 min (4°C, 11,180 g) to obtain the final extracts. Before proceeding to NMR analysis, the methanol in the final extracts was removed by a rotary evaporator (SC110A, Thermo, Germany). The remaining of the evaporated extracts were lyophilized and resuspended in 550 μl Na^+^/K^+^ buffer (0.15 M, 80% D_2_O, 0.01071% TSP, pH 7.40) and cleaned by centrifugation (4°C, 11,180 g, 10 min); 500 μl of each sample was transferred to a 5-mm NMR tube for ^1^H NMR detection.

#### NMR Spectroscopy

NMR spectra were acquired at 298 K on a Bruker AVIII 600 MHz NMR spectrometer (600.13 MHz for proton frequency) equipped with a cryogenic probe (Bruker Biospin, Germany) at 298 K.

For liver samples we used the first increment of the NOESY pulse sequence (RD-90°-t1-90°-tm-90°-acquisition; t1 = 4 μs, tm = 100 ms). A total of 64 transients for each sample were collected into 32 K data points over a spectral width of 20 ppm with a 90° pulse length adjusted to 10.15 ms.

#### NMR Spectral Data Analysis

The free induction decays were multiplied by an exponential window function with the line broadening factor of 1 Hz prior to Fourier transformation. Each spectrum was corrected for phase and baseline deformation manually using Topspin 2.1 (Bruker Biospin) and the chemical shift (TSP at δ 0.00 for liver). The spectral region (0.5–10 ppm for liver) was integrated into bins with a width of 0.002 ppm using AMIX package (v3.9.2, Bruker Biospin). Some unwanted signals, such as water signals (δ 4.59–5.18 ppm) and methanol signals (δ 3.35–3.37 ppm), were removed (15). The range of each integration interval was 0.002 ppm. All data were normalized by the wet weight of each sample.

### GC-FID-MS Analysis of Fatty Acid Composition for Liver Tissue

Liver fatty acids were methylated following the methods described previously with some modifications (16, 17). Briefly, 10 mg of liver sample was mixed with the internal standard (C17:0 fatty acid methyl ester) and methyl esterification reagent for reaction. After extraction with hexane, each sample was dried and resuspended in 100 μl of n-hexane for detection. A DB-225 chromatographic column (10 m long, 0.1 mm internal diameter; 0.1 μm coating thickness; Agilent, USA) was used, and injector port and detector temperatures were both set at 230°C. Two groups of samples were randomly interspersed during the sampling process.

### Statistical Analysis

Data are shown as mean ± standard error of mean (SEM). An independent *t*-test was used to compare fatty acid levels between S and HT groups. A Kruskal-Wallis ANOVA was used for the comparison of the S, NT, H, HT groups, and the Mann-Whitney test was used for *post-hoc* individual group comparisons (Bonferroni correction). Statistical significance was set at *p* < 0.05 for all analyses. Statistical analyses were performed in SPSS 19.0.

## Results

### Mice Showed Lower Body Weight After Hypoxic Training

**Figure 1:**
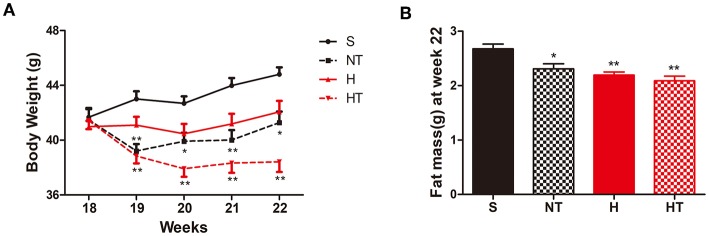
Hypoxic training decreases body weight and fat mass. **(A)** Body weight curves of high-fat diet induced obese (DIO) mice on sedentary (S, *n* = 8), normoxia training (NT, *n* = 8), hypoxia (H, *n* = 10), and hypoxia + training (HT, *n* = 8) for 4 weeks. **(B)** Epididymal and perirenal fat mass in DIO mice after 4 weeks of sedentary (S, *n* = 8), normoxia training (NT, *n* = 7), hypoxia (H, *n* = 8), and hypoxia + training (HT, *n* = 8) treatment at 22 weeks of age. All data are presented as mean ± SEM. *p < 0.05, **p < 0.01, compared with sedentary (S) group mice.

Following 13 weeks of a HFD, mice were randomly assigned to four treatment groups [normoxic sedentary (S), normoxic training (NT), hypoxic sedentary (H), and hypoxic training (HT)] and body weight (BW) was measured at the end of each week during the 4 weeks treatment period (Figure 1A). Over the course of 4 weeks of treatment, hypoxic trained mice had the largest reduction of body weight followed by normoxic trained mice; hypoxic sedentary mice showed the least reductions in body weight while normoxic sedentary mice gained weight. Compared to S mice (control group), HT and NT mice showed consistent significantly lower BW at each of the measurement weeks. In contrast, H mice showed no significant difference in BW compared to S mice and the result was consistent at each of the measurement weeks. Consistent with this, epididymal and perirenal fat mass, measured at week 22, were also significantly lower in the three groups compared to S mice (Figure 1B).

### Mice Have Better Glucose Homeostasis After Hypoxic Training

**Figure 2:**
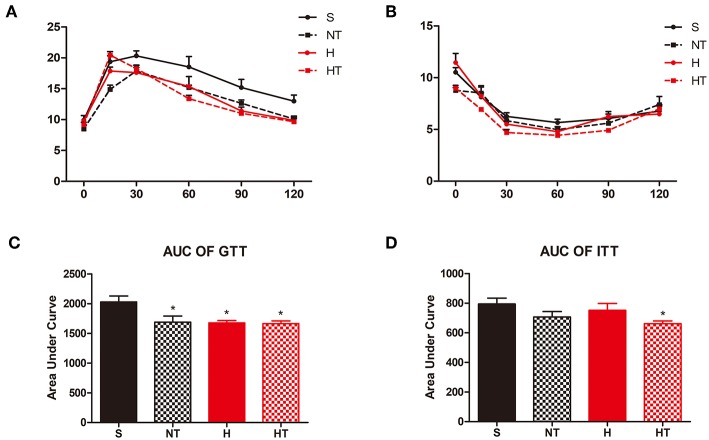
Hypoxic training improves glucose hemostasis of HFD-fed mice. Plots for glucose tolerance tests (GTT, 2 g/kg BW, *n* = 7–8) in overnight fasted mice **(A)** and insulin tolerance tests (ITT, 1 U/kg BW, *n* = 7–8) in mice fasted for 6 h **(B)**, respectively from mice in each group. **(C,D)** AUC values confirmed improvements of glucose tolerance in HT group mice and no difference of insulin tolerance in all the groups. All data are presented as Mean ± SEM, *p < 0.05, compared with sedentary (S) group mice, by two-way ANOVA.

We assessed the effect of hypoxic training on glucose homeostasis and found that hypoxic trained mice had improved glucose tolerance testing relative to normoxic sedentary mice (Figures 2A,C). The same result as the insulin tolerance testing, hypoxic trained mice also had improved insulin tolerance testing relative to normoxic sedentary mice (Figures 2B,D).

### Mice With Hypoxia Training Have Ameliorated Fatty Liver

**Figure 3:**
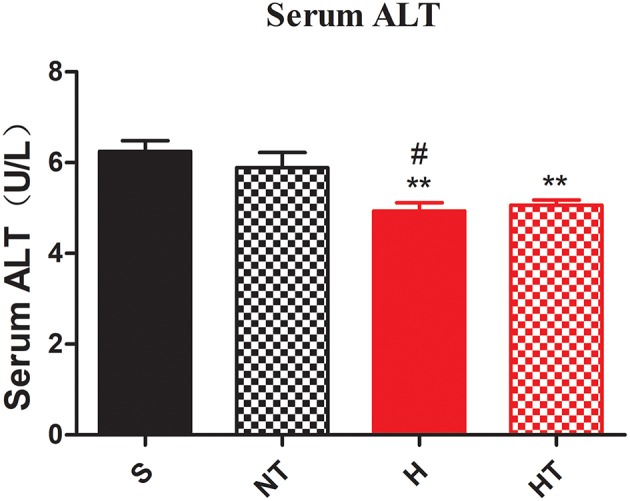
Hypoxic training improves hepatocellular injury of HFD-fed mice. Serum ALT level was decreased in H and HT group (*n* = 7–8). All data are presented as Mean ± SEM, **p < 0.01, compared with sedentary (S) group mice; #*p* < 0.05, compared with normoxic training (NT) group mice, by two-way ANOVA.

**Figure 4:**
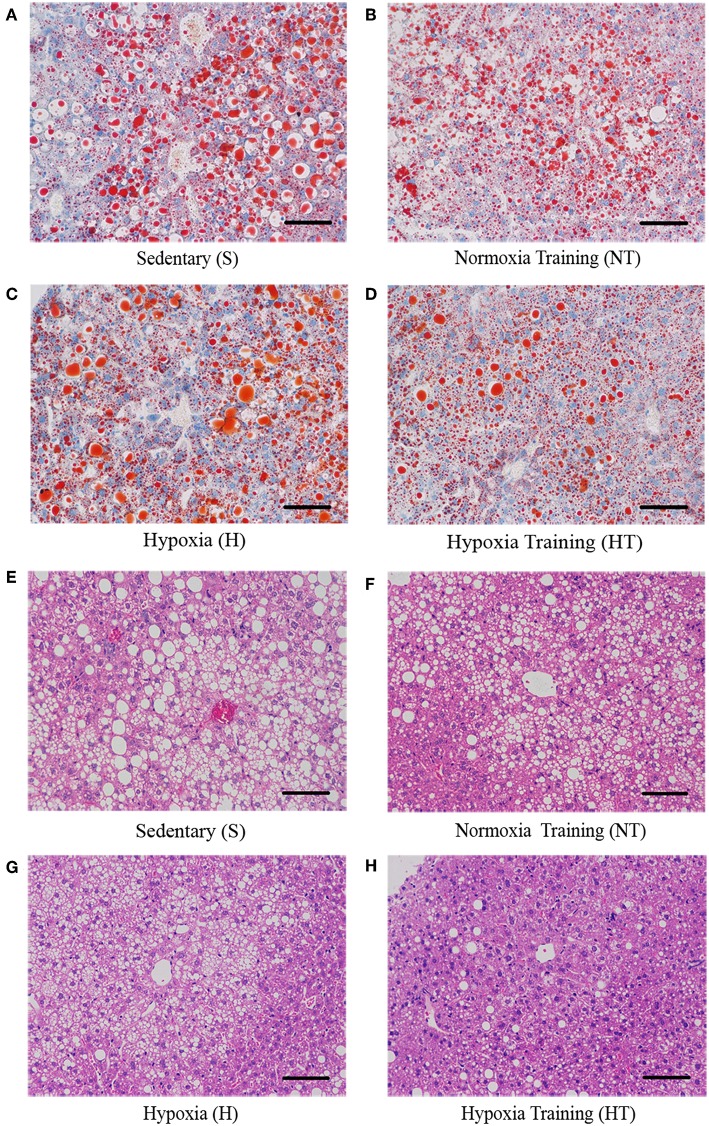
Effect of hypoxic training on the level of lipid droplets in mice liver sections. Representative figures of liver Oil Red O Staining from respectively mice in each group **(A–D)**. Representative figures of liver hematoxylin-eosin staining from respectively mice in each group **(E–H)**. Scale bar: 50 um.

We also asked whether hypoxic training can ameliorate fatty liver or not. As expected, the morphology of liver from hypoxic training group was more reddish than S group (data not shown). Compared to S group, serum Alanine aminotransferase (ALT) levels in HT group were significantly decreased (Figure 3). Concomitant with this, the liver had less lipid droplets in hepatocytes in HT group compared to either S or H groups, though the lipid accumulation was similar to N group (Figure 4).

### Liver Metabolomics Reveals Differential Regulation of Lipid Metabolism by Hypoxic Training

**Figure 5:**
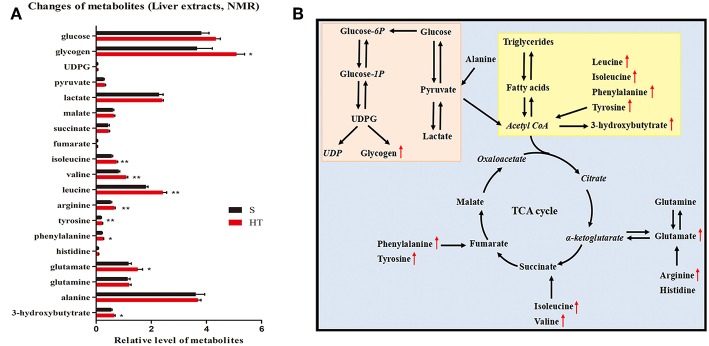
Effects of hypoxic training on glucose and lipid metabolism. Changes of metabolites in response to hypoxic training in mice **(A)**. Data are shown as mean ± SEM, *n* = 8, *p < 0.05, **p < 0.01. **(B)** Summary of metabolic pathways associated with hypoxia training intervention. The italic metabolite represents the undetected metabolite, and the red font is the significantly increased metabolite of Group D vs. Group A. The pink box is glucose metabolism pathway, and the yellow box is lipid metabolism pathway.

Our results from GC-FID-MS (Table 1) showed that, compared to S group, the level of saturated fatty acid C18 in HT group mice was significantly decreased. Furthermore, HT group mice had obviously lower levels of PUFA, n3 PUFA, n6 PUFA, C20:5n3, and C22:6n3 in liver compared with S group. Moreover, in mice livers, hypoxic training resulted in a significant increase in the level of 3-hydroxybutytrate (Figure 5A).

**Table 1:** GC-FID-MS data for fatty acids detected in mice liver.

**Fatty acid**	**S (umol/g)**	**HT (umol/g)**	***p-*value**
C16:0	498.37 ± 51.25	415.27 ± 52.82	0.278
C16:1n7	43.23 ± 5.37	39.95 ± 5.37	0.673
C18:0	96.68 ± 5.05	82.04 ± 4.33	0.0495^*^
C18:1n9	684.54 ± 71.47	561.83 ± 79.33	0.270
C18:2n6	245.35 ± 14.22	215.53 ± 22.68	0.284
C20:1n9	11.81 ± 1.91	8.64 ± 1.41	0.202
C20:3n6	15.73 ± 1.33	12.36 ± 1.56	0.124
C20:4n6	101.25 ± 3.72	95.25 ± 1.99	0.197
C20:5n3	1.99 ± 0.17	1.39 ± 0.08	0.009^**^
C22:6n3	59.80 ± 2.02	51.87 ± 0.89	0.005^**^
C24:0	1.61 ± 0.08	1.59 ± 0.02	0.850
C24:1	0.70 ± 0.12	0.49 ± 0.04	0.137
ToFA	1,761.05 ± 149.70	1,355.80 ± 117.00	0.057
SFA	596.66 ± 55.95	455.86 ± 39.07	0.066
UFA	1,164.40 ± 93.97	899.94 ± 78.20	0.053
MUFA	740.28 ± 78.29	544.33 ± 62.48	0.078
PUFA	424.11 ± 18.34	355.61 ± 16.73	0.017^*^
n3 PUFA	61.79 ± 2.09	53.26 ± 0.95	0.004^**^
n6 PUFA	362.32 ± 16.98	302.35 ± 15.90	0.024^*^
n6/n3	5.87 ± 0.21	5.65 ± 0.18	0.448
PUFA/MUFA	0.60 ± 0.04	0.67 ± 0.04	0.240
PUFA/UFA	0.37 ± 0.02	0.40 ± 0.02	0.255
PUFA/SFA	0.74 ± 0.04	0.79 ± 0.03	0.293
MUFA/UFA	0.63 ± 0.02	0.60 ± 0.02	0.255
SFA%	0.34 ± 0.00	0.34 ± 0.00	0.814
UFA%	0.66 ± 0.00	0.66 ± 0.00	0.815
MUFA%	0.42 ± 0.01	0.40 ± 0.01	0.255
PUFA%	0.25 ± 0.01	0.27 ± 0.01	0.196

Data are shown as mean ± SEM,

* *p < 0.05*,

** p < 0.01.

*ToFA, total fatty acids; SFA, saturated fatty acids; UFA, unsaturated fatty acids; MUFA, monounsaturated fatty acids; PUFA, ployunsaturated fatty acids; PUFA/MUFA, PUFA-to-MUFA ratio; PUFA/UFA, PUFA-to-UFA ratio; MUFA/UFA, MUFA-to-UFA ratio*.

### Hypoxic Training Affects HFD-Induced Glucose Metabolism in Liver

NMR results indicated that hypoxic training for 4 weeks significantly increased the level of glycogen in the liver of HFD-induced obese mice (Figure 5A). Additionally, hypoxic training significantly increased the levels of branched-chain amino acids including isoleucine, valine, and leucine in mouse livers. However, no significant change was found in TCA cycle between HT and S groups. Taken together, our data reveal that hypoxic training promotes liver glucose metabolism in HFD-induced obese mice (Figure 5B).

## Discussion

At present, healthy diet and exercise are recognized as the most safe, effective and economical way to prevent obesity, but the issue that needs to be solved urgently is the increase of appetite (18, 19). Studies have shown hypoxia itself can cause a decrease in appetite and energy intake (20). Till date, epidemiological studies using large scale databases show a negative relationship between habitation in hypoxic conditions and obesity, without clear underlying mechanisms. Recently, it has been reported that O_2_ variations in organic systems may lead to considerable (3%) weight loss and improve metabolic and cardiorespiratory health (21–23). This suggests that sustained hypoxia may benefit obese individuls' weight management (24, 25). In the present study, we have found that hypoxic training has decreased body weight and fat mass of HFD-induced obese mice and has improved glucose tolerance and fatty liver. We have also shown that metabolites related to glucose and lipid metabolism in the liver have been changed after hypoxic training.

In recent studies, intermittent hypoxia has been used as an adjuvant therapy to enhance weight loss in obese patients; additional weight loss was achieved when combined with exercise (26, 27). Human studies have shown that hypobaric hypoxic living conditions have decreased body weight in obese subjects (4). Hypoxia might lead to negative energy balance with weight loss through reducing energy intake and increasing energy expenditure (22). But the mechanism is still to be further studied. One small trial in humans indicate that low intensity exercise training in normobaric hypoxia may lead to more weight loss than normoxic training (21). Furthermore, studies have shown that several weeks of moderate exercise in normoxic conditions resulted in more body weight and fat mass reductions in obese persons compared to the same relative intensity in normobaric hypoxic conditions (23, 28). Animal studies shown that normobaric hypoxia plus exercise training resulted in lower adiposity in HFD Sprague-Dawley rats, and altered the adipose tissue leptin/leptin receptor (24). Our data demonstrate that hypoxic training can decrease body weight and fat mass in mice, which is consistent with the results of previous studies, and suggests that hypoxic training decreases body weight by fat mass loss.

Previous studies have shown that both exercise training and prolonged intermittent normobaric hypoxia have improved glucose tolerance in male Sprague-Dawley rats, and the improvement by hypoxia treatment was significantly greater than that with exercise training alone. (29). Groote et al., found that training in hypobaric hypoxia improved normoxic glucose tolerance in adolescents with obesity (30). In this study, after 4 weeks of hypoxic training, we observed improved glucose tolerance and insulin resistance in HFD-induced obese mice. The improvement of glucose tolerance and insulin resistance by hypoxic training seems to be partly related to the reduction in body weight.

Animal studies have shown that moderate exercise can decrease serum ALT activity, reduce liver injury and fibrosis by inhibiting macrophage infiltration, prevent liver steatosis and effectively improve the pathogenesis of non-alcoholic fatty liver disease in DIO mice (31). In addition, hypobaric hypoxia and intermittent normobaric hypoxia can reduce not only body weight by increasing leptin concentration, but also decrease blood cholesterol, and prevent liver steatosis in obese mice (26). Our current study observed the combined effects of normobaric hypoxic conditions and exercise training on the liver in HFD-induced obese mice. We found that both hypoxia and hypoxic training significantly reduced ALT activity, which suggested that liver injury was alleviated after intervention. Furthermore, liver morphology staining results showed a significant reduction in lipid droplets in hepatocytes in the hypoxic training group. This suggests that hypoxic training can reduce liver lipid accumulation and ameliorate fatty liver in HFD-induced obese mice. These all illustrated that although both hypoxia and normoxic training can partly improve fatty liver, hypoxic training intervenes with the optimal effect.

To further explore how hypoxic training specifically affects liver glucose and lipid metabolism, the current study has applied metabolomics. Our data demonstrated that hypoxic training decreased the levels of saturated fatty acid C18, n3 PUFA and n6 PUFA in liver, and increased the level of 3-hydroxybutytrate. These results suggest that hypoxic training regulates fatty acid metabolism in the liver of HFD-induced obese mice. Studies have shown that branched-chain amino acids could increase glycogen synthesis in liver (32, 33). NMR results indicate that hypoxic training increases the levels of glycogen and branched-chain amino acids in the liver of HFD-induced obese mice. Taken together, our data reveals that hypoxic training promotes liver glucose metabolism in HFD-induced obese mice.

In conclusion, hypoxic training of 4 weeks has reduced body weight, improved glucose tolerance, rescued fatty liver and changed the glucose and lipid metabolites in HFD-induced obese mice. Future studies with exposure to hypoxia are warranted to compare the effects of various exercise modalities/intensities on changes in gait pattern. The transgenic models under or overexpressing genes involved in the oxygen transport system and crucial metabolic pathways can be of valuable interest for future research in this domain.

## Data Availability

The raw data supporting the conclusions of this manuscript will be made available by the authors, without undue reservation, to any qualified researcher.

## Ethics Statement

This study was carried out in accordance with the recommendations of Science Research Ethics Committee at the Shanghai University of Sports (No. 2015013). The protocol was approved by the Animal Care and Use Committee at the Shanghai University of Sports.

## Author Contributions

SG helped conceive the design, performed the NMR and GC-FID-MS analyses, analyzed the data, and wrote the first draft of the manuscript. HT and YH performed other data analyses and helped to draft the manuscript. QY helped conceive the design and supervised experimental trials and training sessions. KZ and C-HK assisted with data analyses and helped draft the manuscript. SH interpreted the study results and edited the manuscript. RW, PC, and TL helped conceive the design, assisted with data analyses, provided funding for the study, and helped draft the manuscript. All authors have read and approved the final version of the manuscript and agree with the order of presentation of the authors.

### Conflict of Interest Statement

The authors declare that the research was conducted in the absence of any commercial or financial relationships that could be construed as a potential conflict of interest.
